# Efficacy of Exosome-Based Therapies for Skin Rejuvenation: A Systematic Review of Human Studies

**DOI:** 10.7759/cureus.104182

**Published:** 2026-02-24

**Authors:** Julio César Flores Rodríguez, Luiz Eduardo Toledo Avelar, Kyuho Yi, Rodrigo Merino Arellano, Jesús Antonio López Rodríguez, Miriam Selene Garza Vargas

**Affiliations:** 1 Aesthetic and Regenerative Medicine, Sociedad Mexicana de Investigación en Medicina Estética (SOMIME), Monterrey, MEX; 2 Aesthetic and Regenerative Medicine, Clínica Aura, Monterrey, MEX; 3 Aesthetic and Regenerative Medicine, Sociedade Brasileira de Cirurgia Plástica (SBCP), Belo Horizonte, BRA; 4 Anatomy, Yonsei University, Seoul, KOR; 5 Plastic Surgery, TecSalud, San Pedro Garza García, MEX

**Keywords:** aesthetic dermatology, exosomes, extracellular vesicles, mesenchymal stem cells (mscs), skin rejuvenation

## Abstract

Exosomes are secreted tiny organelles that are single-membrane enclosed and can perform a broad spectrum of functions upon release, such as the reorganization of extracellular matrix and communication with other cells through the release of signals and chemicals, which play an important role in development, immunity, tissue homeostasis, cancer, and neurodegenerative diseases, among other aspects of human health and disease. This systematic review aimed to evaluate the clinical efficacy and safety of exosome-based therapies for skin rejuvenation in human populations and to identify gaps in the existing evidence. A methodological literature search was performed in databases such as PubMed, Scopus, Cochrane Library, and Web of Science with the Preferred Reporting Items for Systematic Reviews and Meta-Analyses (PRISMA) 2020 guidelines. Clinical experiments that were conducted on humans to assess exosome-based interventions to rejuvenate the aesthetic skin were encompassed. The process of study selection, data extraction, and quality appraisal was done independently by two reviewers. Synthesis of results was narrative, since there was methodological heterogeneity. A total of 19 studies were included, but most of them were not randomized. There was an association between exosome-based interventions and skin hydration, elasticity, wrinkles, pores, pigmentation, and overall appearance improvement in the short term. The majority of the studies reported a positive safety profile of topical application, with the exception of individual reports of risks with off-label injectable use. Exosome-based therapies showed encouraging early clinical effects of skin rejuvenation, and the existing evidence is marred by heterogeneity and lack of follow-up. Rigorous randomized trials and standardized reporting are required.

## Introduction and background

Skin is a highly specialized organ that consists of several structurally and functionally different layers, which, together, offer protection, sensory sensation, and homeostatic control [1]. The development of histological and imaging procedures enhanced the study of the skin's architecture and demonstrated that there is a complicated spatial organization among the keratinocytes, fibroblasts, melanocytes, vascular networks, and immune cells [2]. This complex structure allows the dynamically responsive skin to respond to environmental stressors and trauma. Normal repair mechanisms may, however, be impaired by interference with this well-regulated structure due to trauma, inflammation, or aging [3]. Consequently, structural damage tends to take the form of scarring, changes in pigmentation, or early aging, all of which have functional and psychosocial implications.

Wound healing is a highly controlled biological process that requires inflammation, proliferation, and remodeling of the underlying structure of normal skin. In case of the situation descending to dysregulation, pathological scarring may develop, such as hypertrophic scars, keloids, or atrophic lesions [4]. There is experimental and clinical evidence that the primary cause of abnormal scar formation is failure to restore the normal architecture of the extracellular matrix. In addition, continuing fibroblast stimulation and collagen deposition also worsen tissue hardening and mutilation. In that regard, the molecular determinants of scarless and pathological healing are still a top research focus of dermatologic and regenerative studies [5].

Pigmentary disorders and aging of the skin are other effects of broken cutaneous homeostasis besides scarring. Hypertrophic pigmentation results from the disrupted activity of melanocytes and melanosome distribution and commonly relates to inflammation, exposure to UV radiation, and hormonal factors [6]. Simultaneously, intrinsic and extrinsic aging result in the accumulation of oxidative stress, collagen degradation, and dermal loss of elasticity, which also cause wrinkles and changes in texture [7]. This has led to an increased interest in cell-free therapeutic approaches and regenerative approaches.

Exosomes have become important therapeutic mediators of intercellular communication during the last few years. These nanosized extracellular vesicles (EVs) contain bioactive cargo (proteins, lipids, and nucleic acids) that have the ability to regulate the behavior of the recipient cell [8]. Notably, cellular origin plays a major role in determining the biological activity of exosomes. While exosomes released by stressed or diseased cells can propagate inflammation or endothelial dysfunction [9], exosomes released by mesenchymal stem cells (MSCs) have anti-inflammatory, proregenerative, and immunomodulatory effects.

Exosomes are a specific subtype of EVs, typically ranging from 30 to 150 nm in diameter, formed through the endosomal pathway and released upon fusion of multivesicular bodies with the plasma membrane. In contrast, EVs represent a broader category that includes exosomes, microvesicles, and apoptotic bodies, which differ in size, biogenesis, and biological function. For clarity and consistency, this review uses the term “exosome” when referring specifically to vesicles of endosomal origin, while acknowledging the broader EV classification in accordance with current Minimal Information for Studies of Extracellular Vesicles (MISEV) guidelines [8,9].

When applied to clinical dermatology, the results of stem cell-derived exosome studies suggest that they can be beneficial across a wide range of aesthetic and therapeutic indications, based on their biological characteristics. It has been found that clinical studies benefit from acne scars, skin texture, and photoaging using exosomes as either a single modality or together with energy-based devices and microneedling [10,11]. The new findings can prove that some of them are effective for skin lightening and healing the wound without safety issues, such as live cell therapies [12]. However, inconsistency in isolation procedures, characterization criteria, and reporting of outcomes still hampers cross-study comparability.

Considering such a growing yet diversified body of evidence, a synthesis of research must be conducted rigorously. Standardized methods of reporting and bias assessment are systematic review methodologies, an excellent approach to the evaluation of clinical effectiveness and safety [13]. Additionally, the use of the accepted instruments to evaluate both randomized and non-randomized studies contributes to the validity of the findings comparing the conclusions made based on different design types of studies [14-16].

Given the usage of exosome-based dermatologic regimens in practice, it is of utmost importance that a comprehensive exercise be undertaken to provide clear, methodologically sound, and clinically relevant information that can essentially be utilized to develop an informed practice. In order to assess the effectiveness of therapy regimens, an understanding of the previous body of research in the context of standardized reporting methodologies is vital, ultimately having direct implications for the efforts being undertaken to elucidate the subject as well as provide clear directions regarding how to make such an exercise clinically relevant in terms of the decisions being taken for the treatment of a regenerative intervention. This systematic review aimed to evaluate the clinical efficacy and safety of exosome-based therapies for skin rejuvenation in human populations and to identify gaps in the existing evidence.

## Review

Methodology

Study Design and Reporting Framework

This systematic review was performed as per the Preferred Reporting Items for Systematic Reviews and Meta-Analyses (PRISMA) 2020 guidelines for systematic review and meta-analysis. Following the PRISMA 2020 guidelines helped to systematically find, evaluate, and combine the available clinical evidence on exosome-based therapies for skin rejuvenation.

Eligibility Criteria

The population, intervention, comparison, and outcome (PICO) framework was used to make sure that the qualifying criteria were clear, methodical, and repeatable: population (human adults undergoing aesthetic or dermatologic skin rejuvenation), intervention (exosome-based therapies utilized independently or as supplementary treatments to dermatological procedures), comparator (none, placebo, standard care, or alternative rejuvenation interventions), outcomes (the results showed improvements in wrinkles, elasticity, texture, pigmentation, and safety outcomes). A priori criteria of eligibility were based on a population, intervention, and outcome framework. The inclusion criteria were that the studies had to include human subjects who were undergoing aesthetic or dermatologic skin rejuvenation treatments. Qualified interventions included exosome-based treatments to be used as either individual protocols or as a supplement to other dermatological treatments. Eligible study designs included randomized controlled trials and various types of non-randomized studies involving humans, such as split-face trials, single-group studies looking at results before and after treatment, case series, studies comparing treatments with a placebo, and multigroup non-randomized studies. Only peer-reviewed full-text articles in English were considered. Only studies that were animal or in vitro studies, reviews, editorials, letters, conference abstracts, case reports, or single-patient studies were excluded. Also, we eliminated papers dedicated only to wound healing or scar management without focusing on intended outcomes, as well as commercially driven promotional studies that lacked transparency in their methods, to ensure clinical relevance and scientific rigor.

Information Sources and Search Strategy

An extensive literature review was conducted in several electronic databases, such as PubMed, Scopus, the Web of Science, and the Cochrane Library. The databases were systematically searched for studies published between January 1, 2000, and December 31, 2025, with the final search performed on January 15, 2026. It was a combination of controlled vocabulary and free text keywords on exosomes, skin rejuvenation, aesthetic dermatology, and clinical outcomes. Each database had detailed search strings recorded in it, which are presented in Table 1 to promote reproducibility and transparency.

**Table 1:** Comprehensive search strategies for each database, including Boolean operators, applied filters, and total records retrieved during the systematic search (2000-2025; final search on January 15, 2026)

Database	Search strategy	Filters applied	Records retrieved
PubMed/MEDLINE	(("Exosomes"[MeSH Terms] OR exosome*[Title/Abstract] OR "extracellular vesicle*"[Title/Abstract]) AND ("Skin Aging"[MeSH Terms] OR "skin rejuvenation"[Title/Abstract] OR "skin aging"[Title/Abstract] OR "facial rejuvenation"[Title/Abstract] OR "aesthetic dermatology"[Title/Abstract]) AND (humans[MeSH Terms]))	Case reports; clinical study; clinical trial (phase I-IV); clinical trial protocol; comparative study; controlled clinical trial; multicenter study; observational study; randomized controlled trial; English; humans	113
Cochrane Library	(exosome* OR "extracellular vesicle*") AND ("skin rejuvenation" OR "skin aging" OR "facial rejuvenation" OR photoaging OR "aesthetic dermatology") AND (human* OR patient* OR clinical*)	No additional filters	10
Scopus	TITLE-ABS-KEY (exosome* OR "extracellular vesicle*") AND TITLE-ABS-KEY ("skin rejuvenation" OR "skin aging" OR "facial rejuvenation" OR photoaging OR "aesthetic dermatology") AND TITLE-ABS-KEY (human* OR patient* OR clinical)	No additional filters	138
Web of Science	TS=((exosome* OR "extracellular vesicle*" OR "small extracellular vesicle*" OR "exosome-derived") AND ("skin aging" OR "skin ageing" OR photoaging OR "photo-aging" OR photodamage OR rejuvenat* OR antiaging OR "anti-aging") AND (human* OR patient* OR clinical OR trial OR prospective OR randomized OR randomised OR volunteer* OR "in vivo")) NOT TS=(review OR "systematic review" OR meta-analysis OR "meta analysis" OR mouse OR mice OR rat OR murine OR rabbit* OR porcine OR bovine OR "guinea pig")	Excluded reviews and animal studies	19

Study Selection

**Figure 1:**
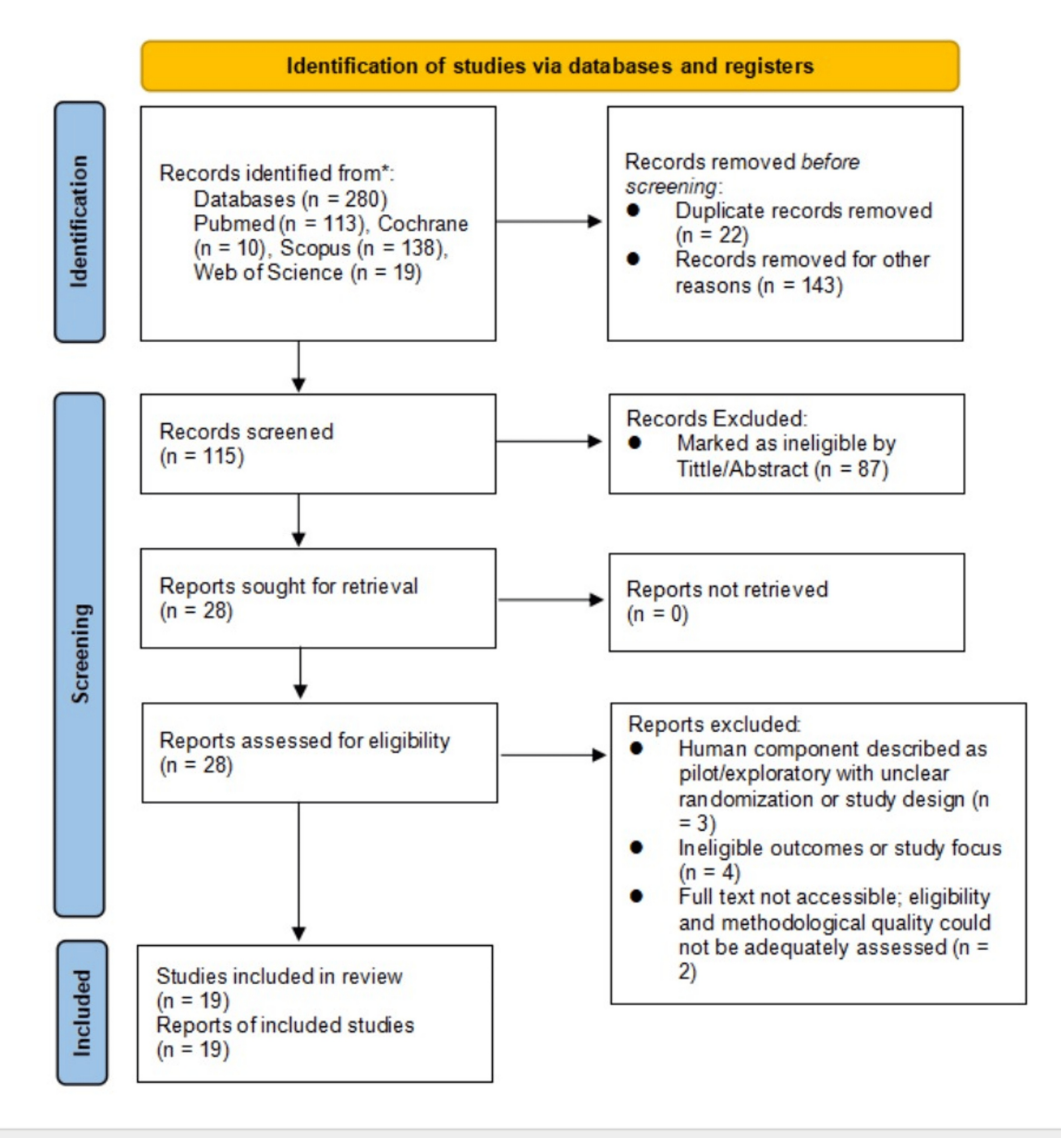
PRISMA flow diagram PRISMA: Preferred Reporting Items for Systematic Reviews and Meta-Analyses Records were identified through database searching (2000-2025; final search on January 15, 2026). Duplicate records were removed using EndNote (Clarivate Analytics), and articles were excluded during title and abstract screening according to predefined eligibility criteria. The diagram was adapted from the official PRISMA 2020 template and manually completed by the authors

The selection of studies was done in two phases. First, titles and abstracts obtained in the database searches were filtered independently by two reviewers to adhere to possible eligibility. Full-text articles were then acquired on studies that met the inclusion criteria or were ambiguous on the aspect of eligibility. The same reviewers engaged in full-text screening. Any differences that arose during the screening steps were resolved through discussion and agreement, and when agreement could not be reached, a third reviewer was consulted. This process of selecting studies in general was recorded via a PRISMA flow diagram that is listed in Figure 1.

Data Extraction and Quality Appraisal

The data extraction was carried out with the help of a standardized data extraction form. Data obtained were research design, sample size, demographics of study population, nature and source of exosome intervention, treatment regimen, measures of outcomes, duration of follow-up, and any adverse effects described. The inclusion criteria were satisfied by 19 studies, and they were incorporated in the final synthesis. Two reviewers extracted data independently to reduce errors and discrepancies. The methodological quality and risk of bias were assessed at the level of the study. The Cochrane Risk of Bias in Non-randomized Studies (ROBINS-I) was used to evaluate randomized controlled trials. The ROBVIS software was used to draw an illustration of the ROBINS-I ROB summary plot. 

Data Synthesis

Quantitative meta-analysis was not done because of the expected heterogeneity in the study designs, intervention protocols, outcome measures, and follow-up periods. Instead, the findings were summarized in a written format, where the results were grouped by the type of intervention and the clinical outcomes reported, making them easier to understand. 

Results

The evidence base was generally non-randomized, with most studies having single-arm pre-post, split-face comparisons, or uncontrolled clinical assessments. The sample sizes varied between 3 and 95, and those studied were normally middle-aged adults, and in some cases, only women were enrolled in the study. The indicators were mainly facial rejuvenation and photoaging, which aimed to treat wrinkles, texture, pores, hydration, pigmentation, and general aesthetic enhancement. There were many different ways the treatments were made, including exosomes from human fat cells, exosomes from platelets with extracts, exosomes from placental stem cells, EVs from a person's own blood, and vesicles from non-human sources like cow's milk, *Lactobacillus*, and plants like Cent. The delivery methods included creams or lotions applied once or twice a day, injections into the skin, and mesotherapy, along with additional treatments after microneedling or radiofrequency microneedling (Table 2).

**Table 2:** Study characteristics ADSC: adipose-derived stem cells; ADV: apple-derived vesicles; AEs: adverse events; BCS: blood cell secretome; BENEV: commercial human adipose mesenchymal stem cell-derived exosome product line; CaHA: calcium hydroxylapatite; ECM: extracellular matrix; EVs: extracellular vesicles; GAIS: Global Aesthetic Improvement Scale; GAGs: glycosaminoglycans; HPE: human platelet extract; IRB: Institutional Review Board; LAEs: *Lactobacillus*-derived artificial extracellular vesicles; LED: light-emitting diode; MSC: mesenchymal stem cells; NRS: numeric rating scale; NTA: nanoparticle tracking analysis; PRFM: platelet-rich fibrin matrix; PRP: platelet-rich plasma; RFMN: radiofrequency microneedling; sEVs: small extracellular vesicles; SEC: size-exclusion chromatography; TEWL: transepidermal water loss; VAS visual analog scale

Study	Country/setting	Design	Sample size (n)	Population (age/sex)	Indication	Exosome source/type	Delivery	Comparator/control	Follow-up duration	Key outcomes measured
Estupiñan et al. [17]	USA; single outpatient dermatology clinical research center (NJ)	Non-randomized, investigator-blinded, split-face, non-inferiority trial	15 enrolled (1 discontinued biopsy)	Age: 44-68; male & female; Fitzpatrick I-IV	Photoaged facial skin (mild-moderate photoaging)	Human adipose mesenchymal stem cell-derived exosomes (BENEV topical products; includes ADSC exosomes; EXO BALM contains ADSC + rose plant stem cell exosomes + other actives)	Topical application postradiofrequency microneedling (RFMN) to one hemiface	PRP/PRFM applied to the opposite hemiface post-RFMN (Selphyl PRFM system)	Photos and clinical grading at baseline, 3 months, 6 months; biopsies baseline/3/6 months in the first 10	Wrinkling, dyschromia, erythema, texture, overall appearance, Griffiths photoaging scale, histology, collagen I/III, GAGs, safety/AEs
Kang et al. [18]	Single-center clinical research organization (country not stated in excerpt)	Non-randomized prospective single-arm (pre-post)	25	40-59 yrs; 18F/7M; mean 49.6±3.3; Fitzpatrick II–IV	Facial photoaging/anti-aging	Autologous blood-derived EVs (SEC isolation; NTA modal ~90 nm)	Single intradermal facial injection (33G; mid-to-deep dermis)	None (no control)	Day 5 and Week 3	Wrinkles, lifting, hydration, TEWL, elasticity, tone/radiance, texture, pores, skin density, subjective satisfaction, AEs
Cho et al. [19]	Single medical institution, South Korea	Retrospective chart analysis (case series)	40	Mean age 41.2±6.3; 32F/8M; Fitzpatrick II-IV	Facial aging/rejuvenation	Commercial exosome-containing skin booster (CUREDOC EXOSOME REPAIR Advanced Solution Skin Booster), 50 mcg/mL	Microneedling (0.25–1.0 mm; 3 passes) + topical/application of exosome booster (3 mL) + LED 5 minutes	None	Followed 8 weeks after the final session (treatment: 4 sessions every 2 weeks)	GAIS from photos, satisfaction (Likert + VAS), intention to repeat/recommend, adverse events
Park [20]	South Korea; dermatology clinic follow-up after procedures done in non-medical settings (beauty salons/non-derm clinics)	Non-randomized case series	4	Adult women: 31, 33, 40, 42 (female only)	Aesthetic skin rejuvenation (off-label injection use)	“Exosome-containing formulation” (exact product/source not specified; unregulated)	Intradermal injection (performed outside medical supervision)	None	Persistent lesions; long course (some cases traced to injections in 2018, 2020, 2021)	Safety outcomes: erythema, nodules, persistent inflammation, scarring; treatment response
Shieh et al. [21]	Taiwan (clinical evaluation; IRB NCKU HREC No. 113-182-2)	Prospective, single-arm, open-label	Hair: 30 (M/F) + Face: 30 (F)	30-65 yrs; facial cohort females only	Skin rejuvenation and hair regrowth	Bio-Pulsed avian MSC-derived sEVs/EVs (primed with *Polygonum multiflorum* extract); topical products (ExoGiov®)	Topical: scalp ampoule regimen 60 days; facial essence twice daily 28 days	No control group (single-arm)	Hair: 60 days (D0/D30/D60); Face: 28 days (multiple timepoints)	Hair: A/T ratio, telogen %, shedding, density, questionnaire. Face: wrinkle depth, pore size, firmness, collagen density, melanin/erythema, gloss, UV spots, irritation erythema model, questionnaires, AEs
Sileo et al. [22]	Human cosmetic testing setting + lab safety assays	Non-randomized clinical	20 (anti-wrinkle study); 20 (lenitive/redness study)	Anti-wrinkle: 41-69 y, both genders; Lenitive: 18-65 y, both genders (Caucasian)	Anti-aging, soothing/redness reduction, and safety	ADVs in a topical formulation (reported as 2% ADV-based formulation for efficacy tests)	Topical	Anti-wrinkle: no stated placebo control (pre-post); Lenitive: hydrocortisone acetate comparator area + irritant model	60 days (anti-wrinkle); 60 minutes post-application (lenitive)	Wrinkle parameters (length/volume/roughness/depth), redness reduction, in vitro safety: genotoxicity, ocular toxicity, irritation/corrosion, sensitization
Wan et al. [23]	South Korea; private aesthetic clinic	Prospective case series (non-randomized)	3	32–55 years; 1 male, 2 females; Fitzpatrick IV	Facial pore reduction, skin texture improvement	Stem-cell-derived exosomes (Exodew; pharyngeal stem cell origin)	Topical exosomes applied immediately after microneedling (0.5 mm)	None	22 weeks	Pore size, skin texture, GAIS (clinician-rated), patient satisfaction, safety
Kang et al. [24]	Korea (industry + university)	Non-randomized pre-post	8 (human efficacy); 14 (microbiome substudy)	Human efficacy: females 40-60 (n = 8); Microbiome: females 20-39 (n = 14)	Skin rejuvenation/anti-photoaging	LAEs from *L. plantarum* OD11	Topical toner (5% LAEs), twice daily	None stated (single arm)	8 weeks (efficacy); 4 weeks (microbiome)	Wrinkles, hydration, redness, texture/roughness, pore area; microbiome diversity/composition; safety observations
Wyles et al. [25]	USA (dermatology clinics)	Prospective non-randomized,	56	Mean age 54 y; adults (sex not specified)	Facial skin rejuvenation	Human platelet-derived extract (HPE) containing platelet exosomes	Topical serum, twice daily	None (single-arm)	12 weeks	Pigmentation, luminosity, color evenness, collagen & elastin histology, safety
Svolacchia et al. [26]	Italy	Non-randomized uncontrolled clinical evaluation	72	Female; 34-68 y (mean 48)	Facial chrono-/photoaging, wrinkles, furrows	ADSC-derived signaling vesicles/exosomes from adipose tissue; conditioned with Skin-B®; ultrafiltered (0.20 µm); “Jaluexos”	Injection (mesotherapy microinjections) into the dermis	None	Follow-ups reported at 15 & 30 days; some figures also show 90 days	Berardesca Scale (satisfaction), NRS (wrinkle/defect severity), VAS, Modified Vancouver Scale (stability/softness/hydration), safety, and flow cytometry markers (CD81/CD146)
Lu et al. [27]	China (clinical + lab setting)	Non-randomized, single-arm clinical study	31	Females, 26-45 y	Skin anti-aging	Bovine milk-derived exosomes (MK-Exo)	Topical application (twice daily)	None (pre–post comparison)	28 days	Skin hydration, elasticity, wrinkle count/area, safety
Nguyen et al. [28]	Vietnam (clinical study + lab work; devices from South Korea/Ireland noted)	Non-randomized, prospective study	3	Adults 30-40 y; sex not clearly stated in excerpt	Skin rejuvenation and photoaging protection	Human adipose-derived stem cell small EVs (ADSC-sEVs) loaded with NR + NAD+ + resveratrol	Topical application on the dorsal hand (daily)	Placebo (0.9% saline) on the opposite hand	8 weeks	Texture, hydration/moisture, elasticity, pore volume, pigmentation/redness/melanin, irritation/s
Chernoff [29]	Not clearly stated (clinical aesthetic setting)	Prospective, non-randomized, multi-arm clinical study	40	35F/5M; age 34-72	Aesthetic skin quality/biostimulation	Placental MSC-derived exosomes (topical emulsion; 1 million in 1 cc stated)	Dermal infusion protocol (exfoliation + NO serum + ultrasound + emulsion + cavitating ultrasound + LED); sometimes followed by CaHA injection	Active comparators: CaHA alone groups (no dermal infusion)	15 & 30 days (Quantificare mentioned; results shown at 30 days)	Quantifiable metrics (wrinkles/pores/evenness/vascularity/oiliness/pigment), satisfaction, AEs
Proffer et al. [30]	USA; single center (Mayo Clinic, Rochester, MN)	Prospective, single-arm, non-randomized longitudinal study	56	Age 40-80 (mean 54 ± 11); 8M/48F; Fitzpatrick I-IV	Facial skin rejuvenation/photodamage & aging	Human platelet extract (HPE), platelet-derived exosome product (“plated” Intensive Repair Serum)	Topical application as part of a standardized twice-daily regimen	No control group (within-subject baseline vs 6 weeks)	6 weeks (±4 days)	VISIA-CR/PRIMOS: wrinkles, erythema, brown spots, luminosity, color evenness, SHS, blinded surgeon photo scoring, participant questionnaires, AEs
Jo et al. [31]	Korea; clinical testing site (Korea Dermatology Research Institute IRB) + in vitro lab work	Human: placebo-controlled study	20 recruited; 16 completed (4 dropouts)	Human trial: Korean women, ~50s average (exact mean not provided)	Skin aging (wrinkles, elasticity, moisture, density, pigmentation)	*Lactobacillus plantarum* extracellular vesicles (LpEVs) isolated from the skin of women in their 20s	Topical application by participants (agent mix; twice daily)	Mannitol 5% placebo vs mannitol 5% + LpEVs	4 weeks (0, 2, 4 weeks)	Wrinkles (Antera 3D), elasticity (Cutometer), dermal density (ultrasound), skin imaging (MARK-Vu), moisture, pigmentation, and in vitro ECM gene/protein markers (MMP-1, COL1A1, FLG, HAS2)
Kerscher et al. [32]	Germany: 6 clinical centers	Prospective, one-armed, multi-center interventional study	95	Women: 30-65 yrs (mean 50.2 ± 8.9)	Facial skin aging/firmness loss	Cell-free blood cell secretome (BCS/ACS) containing cytokines, growth factors, and exosomes	Intra-dermal injections (4 sessions at 0, 2, 4, 6 weeks)	None (no placebo/control arm)	48 weeks	Skin firmness (R0), skin tiring (R3), FACE-Q™, GAIS, patient-perceived age, safety
Park & Shin [33]	Korea; ACE Clinical Research Center (Seoul)	Non-randomized, single-arm pre-post study	Patch test 30; Efficacy 20 (1 dropout from 21)	Patch: adults 35-69 (mean 54); Efficacy: female, 34-63 (mean 50.7 ± 8.9)	Anti-aging skincare/skin rejuvenation	*Centella asiatica* (CICA)-derived extracellular vesicles (EVs)/exosomes; ~100-150 nm; 2.3 × 10⁹ particles/mL; ampoule contains EVs (20,	Topical ampoule twice daily	None (no placebo/control)	Patch: 24 h; Efficacy: 14 days	Patch irritation; pore parameters (Antera 3D); wrinkle depth (Antera 3D); layered hydration (MoistureMeter D; TDC); dermal density (ultrasound)
Wyles et al. [34]	Single-center (Mayo Clinic IRB; Rochester, MN, USA)	Non-randomized prospective longitudinal single-arm	20	Mean age 54 (SD 11); 18F/2M; Fitzpatrick: mostly I–II	Skin rejuvenation/healthy skin aging	Human platelet extract (HPE) containing platelet-derived exosomes/EVs (allogeneic, leukocyte-depleted pooled platelets)	Topical HPE serum twice daily	None (no placebo/control)	12 weeks	Senescence markers (p16INK4a, p21CIP1/WAF1); telomere-associated foci (TAF) & γH2AX; RNA-seq
Chang et al. [35]	Taiwan (HungKuang University site)	Non-randomized clinical study	Treatment 20; Post-hoc placebo 10	Treatment: mean age 36.5; 16F/4M (as stated). Placebo: mean age 40.1; 8F/2M	Facial skin rejuvenation/cosmetic anti-aging	*Centella asiatica* extracellular vesicles (plant-derived EVs) (leaf/petiole + callus vesicle mixture)	Topical serum, 0.1 mL, twice daily for 28 days	EV-free identical base serum (post-hoc, non-concurrent placebo)	28 days (Days 0, 7, 14, 21, 28)	Hydration, elasticity, melanin; wrinkles, redness, pores (VISIA), and irritation/safety

In imaging and biophysical devices such as VISIA (Canfield Scientific, Inc., Parsippany, NJ, USA), PRIMOS (GFMesstechnik GmbH; Teltow, Germany), and Antera 3D (Miravex Limited; Dublin, Ireland), as well as in corneometers, cutometers, tewameters, and ultrasounds, the majority of studies show improvements in hydration, elasticity, wrinkle measurements, pore measurements, and skin texture at baseline over a short follow-up period of 2-12 weeks. Split-face data indicated that topical exosome treatments and platelet-rich plasma had similar effects after radiofrequency microneedling, but there wasn't much formal statistical analysis because the number of participants was small. Other studies also found helpful signs or tissue changes that matched with skin improvement or fewer aging signs, but these couldn't be compared because they looked at different outcomes, times, and quality of reporting (Table 3).

**Table 3:** Summary of clinical outcomes reported across included studies evaluating exosome-based therapies for skin rejuvenation A.U.: arbitrary units; AE: adverse event; ANOVA: analysis of variance; API: advanced probe instrument; BCS: blood cell secretome; CaHA: calcium hydroxylapatite; CI: confidence Interval; DCFDA: 2′,7′-dichlorofluorescin diacetate; ECM: extracellular matrix; EVs: extracellular vesicles; FDR: false discovery rate; GAIS: Global Aesthetic Improvement Scale; GSEA: Gene Set Enrichment Analysis; IHC: immunohistochemistry; IRB: Institutional Review Board; MMP-1: matrix metalloproteinase-1; MSC: mesenchymal stem cells; NRS: numeric rating scale; PRIMOS: phase-shifting rapid in vivo measurement of skin; ROS: reactive oxygen species; SASP: senescence-associated secretory phenotype; sEVs: small extracellular vesicles; SHS: skin health score; TAF: telomere-associated foci; TEWL: transepidermal water loss; UVB: ultraviolet B; VAS: visual analog scale Results are presented as baseline to follow-up changes which were reported, including between-group effects and statistical significance

Study	Outcome domain	Measurement tool	Timepoints	Results (baseline → follow-up)	Between-group effect	Statistical significance (p/CI)	Authors’ conclusion
Estupiñan et al. [17]	Global appearance + wrinkles + dyschromia + erythema + texture	Investigator-rated 5-point scale (0-5) using standardized photos (blinded)	Baseline; 3 months; 6 months	Overall average score baseline 2.12 both sides → PRP 2.70 (3 months), 3.04 (6 months) vs exosomes 2.68 (3 months), 3.14 (6 months)	The exosome side had a slightly greater improvement in wrinkling/erythema/texture; overall appearance & dyschromia were similar	No statistical tests for investigator clinical assessments (small n)	Both treatments improved photoaging features; exosomes were non-inferior clinically
	Photoaging severity	Griffiths Photonumeric Photoaging Scale (0-8)	Baseline; 3 months; 6 months	Baseline 4.5 both sides → 3.1 (3 months) and 3.6 (6 months) both sides; reported % improvement vs baseline: 37% at 3 months, 22% at 6 months	No difference reported	Not reported (clinical scale comparisons descriptive)	Similar improvement with both arms
	Dermal regeneration	Histology: Collagen I/III stains; Alcian blue (GAGs); pathologist scoring + automated image analysis	Baseline; 3 months; 6 months (first 10 participants; 74 samples)	Increased collagen I and GAGs over time in both arms; collagen III total was not different, but stronger staining increased over time	No meaningful arm difference	Paired t-tests/t-tests were used for histology comparisons; there were no significant differences between arms (exact p-values were not provided in your excerpt)	Histology supports dermal remodeling with both; no superiority
	Safety	AE assessment (mild/mod/severe); monitored throughout	Each visit, post-procedure week	No adverse events; expected transient mild pain/erythema/edema/crusting resolved <1 week	N/A	N/A	Topical exosomes are well-tolerated; comparable to PRP side effects
Kang et al. [18]	Wrinkles	Antera 3D CS	Baseline, Day 5, Week 3	Periorbital wrinkle depth: -19.05% at Week 3; nasolabial wrinkle depth: -19.68% at Week 3	N/A (single-arm)	p < 0.05 (Wilcoxon; per caption)	EVs improve wrinkles over 3 weeks
	Lifting/firmness	Antera 3D lifting index/lift volume	Baseline, Day 5, Week 3	Periorbital lift volume -17.55%, Nasolabial lift volume -29.38% at Week 3	N/A	p < 0.05	Improved firmness/lifting
	Hydration	Corneometer CM 825	Baseline, Day 5, Week 3	+22.65% (Day 5), +34.52% (Week 3)	N/A	p < 0.05	Increased hydration
	Barrier function	TEWL via Tewameter TM 300	Baseline, Day 5, Week 3	TEWL -16.17% (Day 5), -25.85% (Week 3)	N/A	p < 0.05	Improved barrier
	Elasticity	Cutometer (R2)	Baseline, Day 5, Week 3	R2 +17.81% (Day 5), +29.76% (Week 3)	N/A	p < 0.05	Increased elasticity
	Texture	Antera 3D (Ra roughness)	Baseline, Day 5, Week 3	Ra -17.11% at Week 3	N/A	p < 0.05	Smoother texture
	Pores	Antera 3D (pore area/volume)	Baseline, Day 5, Week 3	Pore area: -32.35% (Day 5), -46.80% (Week 3); Pore volume: -36.16% (Day 5), -50.62% (Week 3)	N/A	p < 0.05	Pore refinement
	Tone/radiance	VISIA-CR (L-value, radiance intensity)	Baseline, Day 5, Week 3	Tone and radiance significantly improved at Day 5 and Week 3 (exact values not in the excerpt).	N/A	p < 0.05	Brighter/radiant skin
	Dermal regeneration proxy	22 MHz ultrasound skin density	Baseline, Day 5, Week 3	Skin density +10.43% at Week 3	N/A	p < 0.05	Increased density/compactness
Cho et al. [19]	Global aesthetic improvement	GAIS (3 independent evaluators from photos)	Baseline → follow-up (8 weeks after final treatment)	40% very much improved, 45% much improved, 12.5% improved, 2.5% no change → 97.5% improved overall	N/A	Not reported in excerpt	Combination therapy improved facial aging
	Patient satisfaction	Likert + VAS + willingness items	Follow-up	Overall satisfaction: 87.5%; 82% would repeat; 85% would recommend	N/A	Not reported	High acceptance and satisfaction
	Texture/tone (qualitative)	Clinical photos (descriptive)	Follow-up	Photos show improvement in texture/tone (no numeric device outputs provided)	N/A	Not reported	Visible improvements reported
Kang et al. [20]	Safety/adverse events	Clinical observation and photos; treatment records	After injection → dermatology presentation → post-treatment course	4/4 developed persistent inflammatory reactions (erythema, nodules; incomplete resolution; residual scarring common)	N/A	N/A	Off-label intradermal injection of unapproved exosome formulations may cause serious, persistent cutaneous complications; regulation and education are needed
Shieh et al. [21]	Wrinkles	Antera 3D	Day 0, 7, 14, 28	Wrinkle depth ↓ (reported ~7.5% at Day 28)	N/A	Day 14: p = 0.0288; Day 28: p < 0.0001	Topical Bio-Pulsed AMSC-sEVs improved facial aging markers
	Firmness/elasticity	DermaLab Combo (elastometry)	Day 0, 7, 14, 28	Firmness ↑ 14.0% by Day 28	N/A	Day 14 p < 0.0001; Day 28 p < 0.0001	Improved dermal mechanical properties
	Collagen/dermal regeneration	DermaLab Combo (20 MHz ultrasound)	Day 0, 7, 14, 28	Collagen density ↑ 18.2% at Day 28	N/A	p < 0.0001	Suggests dermal matrix improvement
	Pores	Antera 3D	Day 0, 7, 14, 28	Pore area ↓ 8.5% at Day 28	N/A	p < 0.0001	Reduced pore size
	Pigmentation	Mexameter MX18; Antera 3D; VisioFace RD	Day 0 → Day 28	Melanin index ↓ 6.5%; pigmented spots ↓ 5.3%; UV spots ↓ 5.3%	N/A	p < 0.0001 for these reported endpoints	Improved tone/brightness
	Erythema/redness	Antera 3D; irritation model (Chromameter a-value)	Baseline → Day 28; irritation follow-up	Erythema index ↓ 3.9% at Day 28 (p = 0.0101). Irritation model: redness reduction 14.2% vs 9.4% control at 60 min	Internal untreated control in the irritation model only	p < 0.0001 (irritation model); p = 0.0101 (facial erythema)	Mild but significant soothing/anti-inflammatory effect
	Hair outcomes (supportive)	Scalp analyzer, comb test, imaging	D0/D30/D60	A/T ratio ↑ (1.32→1.97); telogen % ↓; shedding ↓ (8.60→4.60 hairs)	N/A	Multiple significant comparisons were reported	Improves hair cycling and reduces shedding
Sileo et al. [22]	Wrinkle length	Antera 3D	T0, T30, T60	Reduced over time (exact baseline values not provided in your excerpt; Table 3 reports change metrics)	No placebo stated	p < 0.05 (reported as significant reduction)	ADV formulation improved wrinkle appearance
	Wrinkle volume	Antera 3D	T0, T30, T60	Reduced over time (Table 3 reports ΔT30-T0 = −0.631; ΔT60-T0 = -0.892)	No placebo stated	p < 0.05	Reduced wrinkle volume
	Roughness (Ra)	Antera 3D	T0, T30, T60	Improved (decreased roughness) by T60 (Table 3 reports ΔT60-T0 = -0.759)	No placebo stated	p ≤ 0.05	Smoother skin surface
	Wrinkle depth	Antera 3D	T0, T30, T60	Small change (Table 3 shows Δ ~0.0005)	No placebo stated	Not significant (as described)	Depth not meaningfully changed
	Skin redness (soothing/lenitive)	Instrumental redness probe	15, 30, 60 minutes after the product	Redness reduced at 60 minutes vs irritant baseline	Compared with the hydrocortisone area (faster effect)	p < 0.05 at 60 min	ADV cream reduces irritation-related redness (slower than hydrocortisone but effective)
Wan et al. [23]	Pore size	Standardized clinical photography; GAIS	Baseline; 12 & 22 weeks	Visible reduction in pore size in all patients; GAIS 4-5 (“very much improved”)	Not applicable	Not reported	Combination therapy improved pore appearance
	Skin texture	GAIS; photographic assessment	Baseline; 12 & 22 weeks	Smoother skin texture observed across the nose and cheeks	Not applicable	Not reported	Enhanced skin texture with sustained improvement
	Patient satisfaction	Self-reported 4-point scale	12 & 22 weeks	High satisfaction (scores 2-3 across all cases)	Not applicable	Not reported	Patients are satisfied with the cosmetic outcome
	Safety	Adverse-event monitoring	Throughout study	No serious adverse events reported	Not applicable	Not applicable	Treatment well-tolerated
Kang et al. [24]	Wrinkles	Antera 3D	Up to 8 weeks	Frontal eye: -11.49%; lateral eye: -20.23%; nasolabial fold: -11.43% by 8 weeks	N/A	Reported as significant for most wrinkle types after 2 weeks (except nasolabial folds)	LAEs improve multiple facial wrinkles
	Hydration	Corneometer	Up to 8 weeks	Improved; plateau noted after ~4 weeks	N/A	Not fully specified in excerpt (improvement described as significant after 4 weeks)	LAEs improve skin hydration
	Redness (inflammation proxy)	Chromameter + Antera 3D	Up to 8 weeks	Redness: -7.18% by 8 weeks; slight early increase, then decrease	N/A	Not fully specified in the excerpt	After acclimation, LAEs may soothe skin
	Texture/roughness	Chromameter + Antera 3D	2 weeks; 8 weeks	-3.78% at 2 weeks; -11.79% at 8 weeks	N/A	Significant improvements stated	LAEs improve texture
	Pore area	Antera 3D	2 weeks onward	Significant decrease after the first 2 weeks	N/A	Significant stated	LAEs reduce pore area
	Pigmentation/color evenness	VISIA-CR, PRIMOS 3D	Baseline → 12 weeks	87.3% subjects reported visible improvement	N/A	p ≤ 0.001	Topical HPE improves facial skin appearance
	Luminosity/skin quality	Participant questionnaires and imaging	Baseline → 12 weeks	Sustained improvement at 12 week	N/A	p ≤ 0.001	Cosmetic benefit maintained
	Collagen remodeling	Masson’s Trichrome + EM	Baseline → 12 weeks	Significant increase in collagen fibril thickness	N/A	p ≤ 0.0001	HPE enhances dermal collagen structure
	Elastin formation	Verhoeff-Van Gieson stain	Baseline → 12 weeks	Increased elastin density	N/A	Reported significant	Supports dermal regeneration
Wyles et al. [25]	Wrinkles/skin aging	VISIA-CR 3D, PRIMOS imaging	Baseline, 12 weeks	Visible improvement in facial skin aging parameters; increased luminosity and color evenness	Not applicable (single-arm study)	p ≤ 0.001	Topical HPE improved overall facial skin appearance after 12 weeks
	Pigmentation	VISIA-CR 3D photography	Baseline, 12 weeks	Sustained reduction in facial pigmentation reported by 87.3% of participants	Not applicable	p ≤ 0.001	HPE demonstrated pigment-reducing effects
	Texture/elasticity (indirect)	Participant questionnaires and imaging	Baseline, 12 weeks	Subject-reported improvement in texture, luminosity, and color uniformity	Not applicable	p ≤ 0.001	Participants perceived a meaningful cosmetic improvement
	Dermal regeneration (collagen)	Histology (Masson’s trichrome), electron microscopy	Baseline, 12 weeks	Significant increase in collagen fibril thickness	Not applicable	p ≤ 0.0001	Topical HPE promoted dermal collagen remodeling
	Safety/tolerability	Clinical monitoring	Throughout study	No serious adverse events; well-tolerated	Not applicable	Descriptive	HPE is safe for topical facial use
Svolacchia et al. [26]	Patient satisfaction/skin firmness & cutaneous relief	Berardesca Scale (0-4)	D0 → D30 (and D90 shown in figure)	Improvement vs baseline (direction: ↑ satisfaction)	N/A	p < 0.0001 (one-way ANOVA)	Treatment improves visible aging signs
	Wrinkle/defect severity	NRS (0-10)	D0 → follow-up (figure indicates improvement during follow-up)	Severity reduced vs baseline (direction: ↓)	N/A	p < 0.05 and p < 0.0001 reported	Exosome nanofiltrate reduces wrinkle/defect severity
	Global skin appearance (stability/softness/hydration)	Modified Vancouver Scale (MVS)	D0 → D15/D30	Improvement vs baseline (direction: ↑)	N/A	p < 0.0001 (one-way ANOVA)	Skin quality improves after treatment
	Biological confirmation of vesicles	Flow cytometry (EV kit; CD81/CD146)	Post-processing verification	Reported ~450 million CD81+ secretory vesicles (standardized sampling)	N/A	Not stated	Protocol yields vesicles with exosomal markers
Lu et al. [27]	Skin hydration	Corneometer CM825	Day 2, 14, 28	+5.6% at Day 28 (overall); +7.96% in age 36-45	Not applicable	p < 0.05	MK-Exo improves skin moisturization
	Elasticity	Cutometer (F3/F4, R2)	Day 14, 28	F3/F4 +6.33%; R2 +7.24% at Day 28	Not applicable	p < 0.01	Improved skin elasticity
	Wrinkle count	PRIMOS CR	Day 14, 28	-4.99% at Day 28	Not applicable	p < 0.001	Reduced wrinkle number
	Wrinkle area	PRIMOS CR	Day 14, 28	-9.59% at Day 28	Not applicable	p < 0.001	Reduced wrinkle severity
Nguyen et al. [28]	Photoaging protection (oxidative stress)	DCFDA ROS assay in UVB-exposed HaCaT	After an hour of treatment + UVB	Loaded sEVs reduced ROS by 38.3% vs sEVs 13.3% vs compounds 18.5%	Not applicable (in vitro arms)	p < 0.05 stated generally	Loaded sEVs show synergistic protection
	Hydration/moisture	GPSkin Barrier + API-100	Every 2 weeks, endpoint 8 weeks	Hydration/moisture improved (reported +104% hydration and elasticity-see below note)	The placebo hand had minimal change	p < 0.05 (t-test reported)	Skin hydration improved
	Elasticity	API-100 Skin Analyzer	Every 2 weeks, endpoint 8 weeks	Elasticity increased by 104%	Placebo minimal change	p < 0.05 (reported)	Elasticity improved
	Texture/pore	Antera 3D + API-100	Every 2 weeks, endpoint 8 weeks	Texture smoother; mean pore volume reduced 51%	Placebo minimal change	p < 0.05 (reported)	Rejuvenation effect on texture/pore
	Pigmentation/redness/melanin	Antera 3D + API-100	Every 2 weeks, endpoint 8 weeks	Less pigmentation/redness (qualitative); melanin decreased by API-100 but inconsistent vs Antera	Unclear	Mixed/inconsistent	Whitening effect uncertain
Chernoff [29]	Wrinkles, pores, evenness, vascularity, oiliness, pigment, texture	Quantificare 3-D photo documentation/skin analysis	Baseline → 15 days → 30 days	Improved in all groups (dermal infusion alone and CaHA alone)	Dermal infusion “primer” + CaHA appeared faster/more enhanced than CaHA alone (qualitative claim)	Not reported	Dermal infusion improves absorption; adding CaHA after priming yields faster enhanced skin quality
Proffer et al. [30]	Global skin health	Skin Health Score (SHS; composite)	Baseline vs 6 weeks	Mean delta +224.2 ± 112.8 (top quartile responders n = 14)	N/A (single-arm)	p ≤ 0.0001	Topical platelet exosomes improved overall skin health at 4-6 weeks
	Redness/erythema	VISIA-CR RBX erythema fractional area	Baseline vs 6 weeks	-2.39 ± 2.68 (top quartile n = 14)	N/A	p = 0.005	Reduced redness
	Pigmentation/brown spots	VISIA-CR brown spot fractional area	Baseline vs 6 weeks	-0.0161 ± 0.005 (top quartile n = 14)	N/A	p ≤ 0.0001	Reduced melanin/brown spots
	Luminosity	VISIA-CR luminosity (relative units)	Baseline vs 6 weeks	+5.42 ± 1.36 (top quartile n = 14)	N/A	p ≤ 0.0001	Improved brightness
	Color evenness	VISIA-CR color evenness (relative units)	Baseline vs 6 weeks	+0.071 ± 0.03 (top quartile n = 14)	N/A	p ≤ 0.0001	Improved evenness
	Wrinkles (forehead)	VISIA-CR 3D PRIMOS wrinkle fractional area	Baseline vs 6 weeks	-0			
Jo et al. [31]	Wrinkles (periocular)	Antera 3D (indentation index, A.U.)	0, 2, 4 weeks	-8.9% at 2 weeks; -15.89% at 4 weeks vs baseline (LpEV group); no change in placebo	Placebo shows no meaningful change (reported descriptively)	p-values not clearly provided in the excerpt for this endpoint	LpEVs suppress wrinkle formation
	Elasticity	Cutometer MPA 580	0, 2, 4 weeks	+14.76% at 2 weeks; +27.07% at 4 weeks (LpEV)	Placebo not improved as much (direction reported)	Not clearly shown here	LpEVs improve elasticity
	Moisture/hydration	(Instrument not fully named in excerpt)	0, 2, 4 weeks	+10.79% at 2 weeks; +21.40% at 4 weeks (LpEV); placebo “no significant effect”	LpEV better than placebo	Not clearly shown here	LpEVs increase moisture
	Dermal density	Ultrasound (DermaLab Skin)	0, 2, 4 weeks	Density increased in both: LpEV +39.30% vs placebo +15.19% (rate)	LpEV greater increase	Not clearly shown here	LpEVs enhance skin density
	Pigmentation	MARK-Vu imaging and visual reading	0, 2, 4 weeks	Pigmentation decreased at 2 weeks & 4 weeks in LpEV; the placebo had less/no change (reported)	LpEV better	Not clearly shown here	LpEVs reduce aging-related pigmentation
Kerscher et al. [32]	Skin firmness	Cutometer® (R0)	0, 12, 24, 48 weeks	0.40 → 0.36 mm (Week 24); 0.37 mm (Week 48)	N/A	p < 0.0001 (Week 24)	BCS significantly improves skin firmness
	Skin tiring	Cutometer® (R3)	0, 12, 24, 48 weeks	0.45 → 0.40 mm (Week 24); 0.41 mm (Week 48)	N/A	p < 0.0001 (Week 24)	BCS increases resistance to skin fatigue
	Patient-reported skin satisfaction	FACE-Q™	0, 12, 24, 48 weeks	39.6 → 59.8 (Week 12); sustained to Week 48	N/A	p < 0.0001	Sustained improvement in perceived skin quality
	Facial appearance/psychosocial	FACE-Q™	0, 12, 24, 48 weeks	Significant improvement across domains	N/A	p < 0.0001	Improved facial satisfaction & well-being
	Global aesthetic improvement	GAIS (physician & patient)	6-48 weeks	90.5% physician-rated improvement (Week 6)	N/A	p < 0.001	Objective and subjective improvement
	Perceived age	FACE-Q™ Age appraisal	0, 24, 48 weeks	1.68 years younger at Week 48	N/A	p < 0.0001	Patients perceive a younger facial age
Park & Shin [33]	Pores	Antera 3D	Day 0 → Day 1 (single use)	Mean pore area 0.1378 → 0.1131 mm² (-17.9%); pore density 32.83 → 24.00 ea/cm² (-26.9%); total pore volume 0.8350 → 0.5005 mm³ (-40.1%)	N/A	p < 0.05	Rapid pore-refining effects after a single application
	Texture/roughness	3D imaging output	Day 0 → Day 14	Surface roughness -9.0%	N/A	p < 0.05	Improved skin surface texture
	Wrinkles	Antera 3D	Day 0 → Day 14	Wrinkle depth reduction 7.8-18.8% (forehead -7.8%; glabella -11.3%; crow’s feet -11.7%; nasolabial folds -18.8%; neck -9.5%)	N/A	p < 0.05	Significant multisite wrinkle reduction after 2 weeks
	Hydration (layered)	MoistureMeter D (TDC)	Day 0 → Day 1 (single use)	+7.9% at 0.5 mm (40.4 → 43.5); +6.5% at 1.5 mm (37.3 → 39.8); +4.5% at 2.5 mm (32.3 → 33.77)	N/A	p < 0.05	Significant hydration gains across depths
	Dermal regeneration/structure	Ultrasound (reported as dermal density)	Day 0 → Day 14	Dermal density 21.31 → 24.01 (+12.67%)	N/A	p < 0.05	Increased dermal density after 2 weeks
Wyles et al. [34]	Senescence signaling	IHC + digital quantification (Aperio/QuPath) for p16INK4a, p21CIP1/WAF1	Baseline → 12 weeks	High-baseline subgroup: reduction in high p16INK4a dermal senescent cells	N/A	p = .02	Topical HPE reduces senescence signaling, especially in high-senescence baseline individuals
	Telomere damage/genomic instability	Immuno-FISH for TAF (telomere + γH2AX overlap), ImageJ quantification	Baseline → 12 weeks	Decrease in TAF per nucleus	N/A	p = .03	Topical HPE reduces senescence-associated telomere damage
	Inflammation (SASP)	RNA-seq (DESeq2 + GSEA), subgroup analysis	Baseline → 12 weeks	In high-senescence baseline patients: ~40% reduction in proinflammatory SASP (reported as reduction)	N/A	Not clearly specified in excerpt (RNA-seq uses FDR q < 0.25 for pathway-level GSEA)	Suggests modulation of proinflammatory senescence phenotype
	Dermal regeneration/ECM remodeling	RNA-seq pathway enrichment (Reactome/GSEA)	Baseline → 12 weeks	Upregulation of ECM remodeling pathways (collagen, proteoglycans, elastic fibers) and keratinization pathways	N/A	Pathway significance by FDR q < 0.25 (GSEA threshold described)	Suggests a proregenerative remodeling response
Chang et al. [35]	Hydration	Corneometer® CM825	Day 0, 7, 14, 21, 28	↑ 14.6-21.2% improvement by Day 28 (range across regions)	Treatment improved; placebo, no significant change	Within-group: p < 0.0001; treatment vs. placebo on Day 28 significant for most regions	Cica EV serum improved facial hydration over 28 days
	Elasticity (R²)	Cutometer® (Dual MPA580)	Day 0, 7, 14, 21, 28	↑ 12.0-12.5% by Day 28 (across regions)	Treatment > placebo	Within-group: p < 0.001/p < 0.0001; vs placebo significant on Day 28	Cica EV serum improved elasticity
	Pigmentation (melanin)	Mexameter® MX18	Day 0, 7, 14, 21, 28	↓ 9.2-11.3% melanin by Day 28 (lower scores = less melanin)	Treatment > placebo	Within-group: p < 0.0001; vs placebo significant Day 28	Cica EV serum reduced melanin measures
	Wrinkles	VISIA® (percentile/score approach	Day 0, 7, 14, 21, 28	↑ 32.9-34.8% “wrinkle improvement” by Day 28	Treatment > placebo	Within-group: p < 0.001/p < 0.0001; vs placebo Day 28 significant	Cica EV serum reduced wrinkles (instrumental)
	Redness	VISIA® RBX	Day 0, 7, 14, 21, 28	↑ 26.3-34.0% “redness improvement” by Day 28	Treatment > placebo	Within-group: p < 0.001/p < 0.0001; vs placebo Day 28 significant	Cica EV serum reduced redness
	Pores	VISIA®	Day 0, 7, 14, 21, 28	↑ 40.6-41.3% “pore improvement” by Day 28	Treatment > placebo	Within-group: p < 0.0001; vs. placebo, Day 28 significant	Cica EV serum reduced pores

The majority of managed clinical trials did not report any severe adverse effects and only reported temporary local reactions like erythema, edema, dryness, or mild discomfort, especially during such procedures as microneedling or injections. On the other hand, one case study found that using unregulated intradermal injections of poorly defined exosome products led to long-lasting lumps and scars, which points to the risks when the product quality and medical supervision are not good enough (Table 4).

**Table 4:** Summarizes safety reporting across included studies, including total adverse events, local reactions, serious adverse events, withdrawals related to adverse events, suspected immune or infectious reactions, adverse event collection methods, and overall safety conclusions. AE: adverse event; SAE: serious adverse event; ADSC: adipose-derived stem cell; sEVs: small extracellular vesicles; ADV: apple-derived vesicles; BCS: blood cell secretome; MK-Exo: milk-derived exosomes; VAS: visual analog scale; OECD: Organization for Economic Cooperation and Development; THP: human monocytic cell line used for sensitization testing

Study	Total AEs (n/%)	Local reactions (erythema/edema/pain)	Serious AEs	Dropouts due to AEs	Infection/immune reactions	How AEs were collected	Safety conclusion
Estupiñan et al. [17]	0 AEs reported	Expected transient mild pain, erythema, edema, light crusting; resolved within 1 week	None	None (1 withdrew consent for biopsies, not AE)	None reported	The investigator assessed/documented at visits; periodic review for study continuation	Both arms were well-tolerated; topical exosomes appear safe in this setting
Kang et al. [18]	0/0% reported	None clinically significant reported	None	None	None reported	Dermatologist assessment each visit + participant questioning (active follow-up)	Considered safe over 3 weeks in this sample
Cho et al. [19]	Not reported as total count; AEs described	Erythema 85% (resolved 24-48 hours); edema 65% (resolved within an hour); pain VAS 3.2 ± 1.1	None	Not reported	None reported	Chart documentation + clinician observation	Transient, mild AEs; no serious reactions
Park [20]	4/4 (100%) severe local AEs	Persistent erythema, swelling/warmth, nodules, suspected granulomatous inflammation, residual scarring; some developed atrophic scarring after steroid injections	No systemic SAEs reported; clinically severe local complications requiring repeated procedures	1 case discontinued treatment and self-managed	Immune/inflammatory mechanism suspected; product contamination/excipients discussed as possible contributors (no histology)	Retrospective case documentation from dermatology clinic; patient history of off-site injection; photos	Intradermal injection of cosmetic exosome products (unregulated/off-label) can cause serious and persistent skin complications
Shieh et al. [21]	None reported	None reported	None	None reported	None reported	“Monitored throughout”; compliance and AE monitoring stated (details limited)	Appears well-tolerated in these cohorts
Sileo et al. [22]	Not reported for the 60-day anti-wrinkle use in your excerpt	Not reported	Not reported	Not reported	Not reported	Not clearly stated for human use	Human AE reporting is unclear in the excerpt
Wan et al. [23]	In vitro safety battery	No genotoxicity (Ames negative); no corneal toxicity; non-corrosive; non-irritant; non-sensitizing (THP oSens described)	N/A	N/A	N/A	OECD guideline assays	ADVs show a strong preclinical safety profile for topical use
Kang et al. [24]	0/0%	Mild, transient erythema, edema, and minor bleeding (resolved within 24-72 hours)	None	None	None reported	Active monitoring during and after each session	Combined microneedling and exosome therapy appears safe and well-tolerated
Wyles et al. [25]	Not clearly quantified in the excerpt	Mild transient redness increase early possible; no severe adverse effects reported	None reported	Not reported	None reported; authors mention possible rare mild reactions to non-human derivatives	Not clearly specified (likely active monitoring during visits + questionnaire)	Appears generally safe in small short-term topical use; AE reporting detail is limited
Svolacchia et al. [26]	Not specifically quantified	Mild, transient topical reactions only	None	None	None reported	Active monitoring + participant questionnaires	Well-tolerated; no serious safety concerns
Lu et al. [27]	Not quantified; stated none recorded	Not detailed	None reported	Not reported	None reported	Unclear (appears to be clinical follow-up; not explicitly “active AE surveillance”)	Reported as extremely safe, but AE capture/reporting lacks detail
Nguyen et al. [28]	0/0%	None reported	None	0	None observed	Active monitoring and patch testing	MK-Exo demonstrated good cutaneous safety
Chernoff [29]	0 reported	No itching/redness/swelling/rash/blisters/peeling, etc. in 3 volunteers	None reported	0	None reported	Active checks at baseline, days 1/3/7 first week, then every 2 weeks	Topically loaded ADSC-sEVs appeared safe in this small sample
Proffer et al. [30]	0 reported	None reported	None	0 lost to follow-up	None reported (no hypersensitivity/allergic events)	Not clearly specified	Appears well-tolerated in short follow-up (≤30 days)
Jo et al. [31]	12/56 (21.4%) reported side effects	Dryness 9/56 (16.1%), most common; also monitored redness/irritation/pain/swelling	None reported	0	No allergic or irritant contact dermatitis reported	Monitoring at baseline & 6-week visit + participant reporting	Safe and well-tolerated over 6 weeks; mostly mild dryness
Kerscher et al. [32]	Not clearly reported in the excerpt	Not clearly reported	Not stated	4 dropouts (reason not provided here)	Not stated	Not stated	Safety reporting is insufficient in the provided text (needs a full AE section/details)
Park & Shin [33]	36/380 injections (9.5%)	Hematoma, redness, swelling, pain, urticaria (mostly mild)	None	0	None reported	Active monitoring throughout the study	BCS injections were safe and well-tolerated
Wyles et al. [34]	Patch test: 0/30 irritation (index 0.00). Efficacy: no adverse reactions reported/observed	None in patch test; none reported during 2-week use	None reported	0 (one dropout due to noncompliance, not AE)	None reported	Dermatologist grading at 30 minutes & 24 hours post patch removal; monitoring during use	Product classified as non-irritant and well-tolerated
Chang et al. [35]	Not reported in excerpt	Not reported	Not reported	Not reported	Not reported	Not reported	Safety conclusions cannot be fully extracted from the provided text (article may report elsewhere)
	0 reported	Patch test negative; no irritation/allergy reported	0	0	Not reported/none indicated	Patch test screening and ongoing monitoring during visits	Well-tolerated over 28 days; no safety signals reported

**Figure 2:**
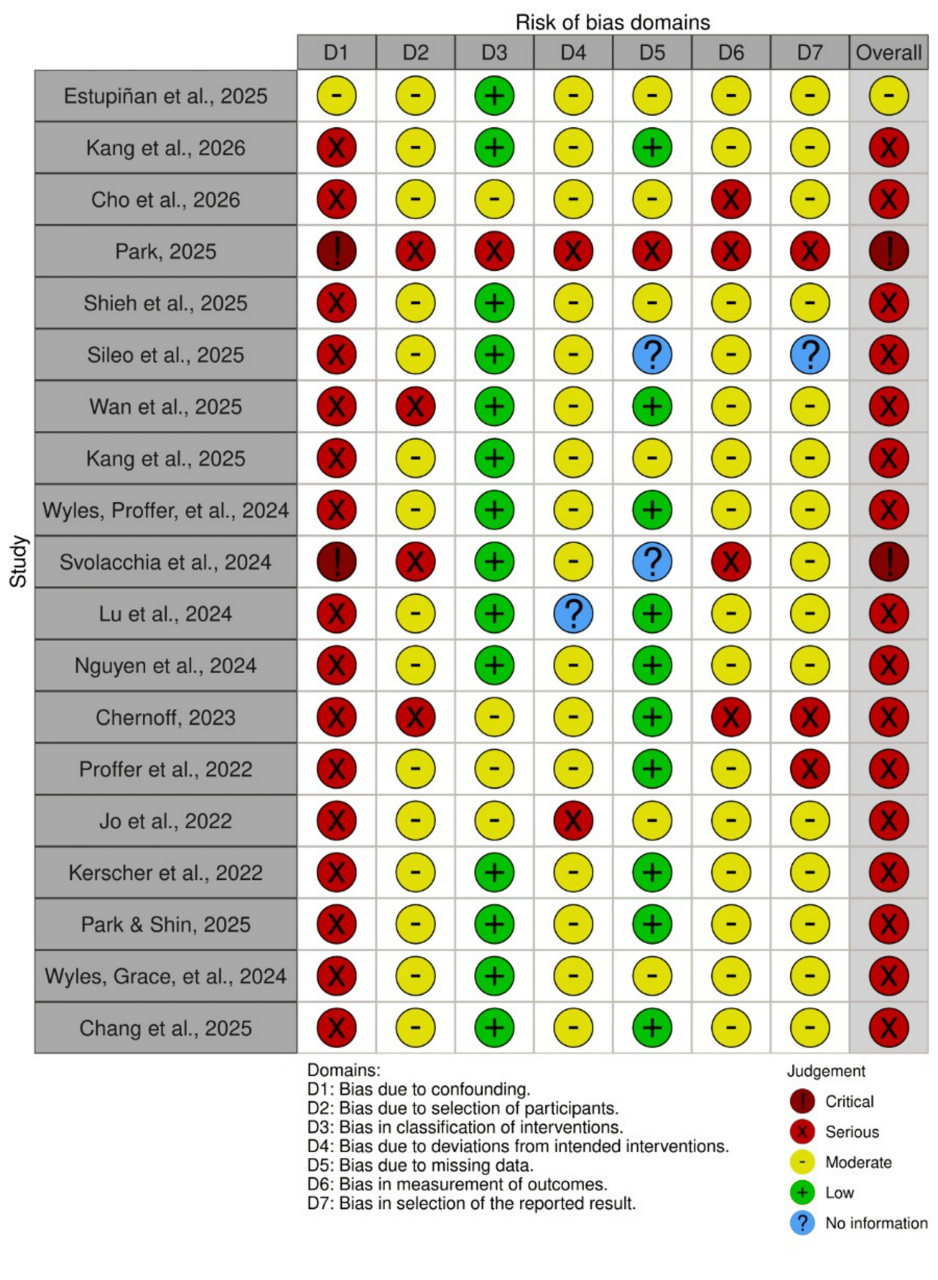
ROBINS-I quality appraisal robvis ROBINS-I: risk of bias in non-randomized studies-of interventions; robvis: risk-of-bias visualization Adapted/reproduced from [17-35], with permission

Figure 2 illustrates the bias risk for each included domain in the research. Most studies showed a moderate to high risk of bias, mainly due to factors that could confuse the results and how participants were chosen, but bias in how interventions were classified, and outcomes measured was usually low. The overall quality of the methods varied, indicating that the current clinical evidence has certain limitations. 

Discussion

Main Findings

This systematic review has summarized current human clinical data on exosome-based skin rejuvenation treatments and found consistent short-term benefits for different beauty aspects. In the studies included, using exosomes as part of skin treatments was associated with better results in hydration, elasticity, wrinkles, pore size, skin color, and overall skin quality. These effects were the most common during the two- to 12-week follow-up, and they were assessed by a combination of validated imaging platforms, biophysical instruments, and investigator-rated scales. Importantly, studies that looked at both sides of the face found that treatments using exosome-containing products worked just as well as traditional methods like platelet-rich plasma when combined with microneedling.

In terms of safety, the majority of regulated clinical studies depicted excellent tolerability rates, and the adverse events were mild, transient local reactions. Nevertheless, the review also found clinically significant complications associated with the off-label, intradermal injection of ill-defined exosome products delivered without medical guidance, illustrating the significance of product standardization and clinical governance. Generally, the results indicated that exosome-based interventions were an exciting but immature modality in aesthetic dermatology; their emerging results were promising but limited by methodological heterogeneity, a lack of long-term data, and a lack of uniformity in reporting standards across studies.

Comparison With Previous Literature

The overall results of this review supported earlier studies that showed the healing benefits of exosomes from mesenchymal stem cells for skin use. Enhanced wound healing and skin quality after topical application of human MSC-derived exosomes in the aftermath of the aesthetic sector have been reported in clinical case series and small prospective studies in the past, supporting the role of paracrine-mediated repair in this review [36]. Likewise, split-face trials using adipose-derived stem cell exosome solutions with microneedling have shown significant improvements in wrinkles, elasticity, and texture compared to baseline and placebo-treated sites, which is consistent with the outcome patterns observed [11].

Evidence from acne scar and pigmentation-focused studies further facilitated the applicability of exosome therapies to a wider range of dermatology indicators. Exosomes made from mesenchymal stem cells might be a beneficial alternative to cell-based treatments for acne scars because they can help regenerate skin with fewer side effects than other types of cell implants [37]. At the same time, studies that randomly tested adipose-derived exosomes on one side of the face showed significant improvements in skin brightness and evenness of tone, which were better than the pigmentation results found in other research included in this review [38].

These clinical observations have been supported by mechanistic evidence in preclinical systematic reviews and meta-analyses. The animal studies show that small EVs made from MSCs help wounds heal better by increasing collagen production, improving blood vessel growth, and adjusting the immune response, which results in better tissue quality and less scarring [39,40]. These biological effects are also seen in other types of tissue, like corneal scarring, where MSC exosomes help reduce inflammation and adjust the immune system, promoting tissue healing [41].

Simultaneously, the exosome literature echoed the methodological issues articulated in the review. The amount, cleanliness, and effectiveness of vesicles can vary depending on the method used for their isolation, such as tangential flow filtration, ultracentrifugation, and size exclusion chromatography, leading to different outcomes in clinical settings [42-44]. Cross-study comparisons are even more complicated due to differences in characterization and quantification techniques, including nanoparticle tracking analysis [45,46]. All these reasons point to the need for consistent methods in making, testing, and reporting exosomes to ensure that the positive effects of exosome-based skin rejuvenation treatments can be reliably repeated and used in everyday medical practice.

Implications of the Findings

The results of this systematic review had significant clinical and research implications for aesthetic and regenerative dermatology. All the evidence showed that a treatment using exosomes could be a useful, non-cell method to improve various skin qualities, like moisture, flexibility, color, and the look of wrinkles. In clinical settings, using topical exosome preparations along with microneedling or energy-based systems appeared to have strong short-term benefits and a good safety record when done in a controlled way. Exosome-based products could complement existing aesthetic procedures, but they shouldn't take their place. The review also pointed out the important role of treatment context, product characterization, and delivery mode in influencing outcomes. To the clinicians, the findings have highlighted the importance of selecting patients carefully, following certified formulations, and avoiding off-label injectable use. The results showed that paracrine signaling plays a key role in skin regeneration and pointed out the chance to standardize exosome-based treatments in beauty practices.

Limitations and Future Recommendations

When interpreting the findings of this review, it is important to consider several limitations. To start, most of the studies included were not randomized and only looked at one group with small numbers of participants, which made it challenging to draw clear cause-and-effect conclusions and increased the chances of bias in the research. Secondly, the studies varied greatly in where the exosomes came from, how they were isolated and prepared, the amounts used, how they were given, what results were measured, and how long they followed up, which made it impossible to combine the results for a thorough analysis. Third, the way outcomes were reported varied a lot, as most studies used short-term indicators, personal satisfaction ratings, or simple comparisons of results without proper statistical analysis. The reporting on safety was inconsistent; many studies vaguely reported adverse events and lacked active surveillance methods. Notably, long-term safety and durability are not adequately defined.

Future studies are encouraged to conduct more effective randomized controlled trials with sufficient sample sizes, standardized outcome measures, and extended follow-up periods to determine durability and delayed adverse events. The exosome manufacturing, characterization, and reporting standards have to be harmonized to enhance reproducibility. It is also suggested that comparative trials with proven regenerative modalities, such as platelet-based therapies, be done. Lastly, regulatory control and clinician training ought to be intensified in order to curb the risks related to unregulated or off-label injectable exosome products.

## Conclusions

Overall, this systematic review revealed that exosome-based treatment had promising short-term effects in various areas of skin rejuvenation, such as hydration, elasticity, texture, pigmentation, and wrinkle appearance. Topical utilization or interventions as adjuncts during conventional aesthetic practice proved to be tolerable in most cases in controlled clinical environments. However, methodological heterogeneity, limited follow-up, and unstable safety reporting still constrain the existing clinical evidence. Although exosome-based skin rejuvenation is a fast-moving and potentially transformative area of research, it will be premature until strong clinical trials and standardized frameworks are established before mass clinical adoption can be supported.
